# Pathology of Grief

**Published:** 1891-09

**Authors:** 


### Pathology of Grief.

That severe mental distress or fright sometimes produces physical disease, and occasionally even death, is an admitted fact, although the way in which it acts has hitherto been but little studied. In order in some measure to supply the deficiency in our knowledge regarding this matter, Dr. G. Bassi has recently made a number of observations on animals which apparently died in consequence of capture. Birds, moles, and a dog which had succumbed to conditions believed by Dr. Bassi to resemble those known amongst human beings as acute nostalgia and "a broken heart" were examined post-mortem. Generally there was hyperæmia, sometimes associated with capillary hemorrhages of the abdominal organs, more especially of the liver, also fatty and granular degeneration of their elements, and sometimes bile was found in the stomach with or without a catarrhal condition. The clinical symptoms were at first those of excitement, especially in the birds, these being followed by depression and persistent anorexia. The theory suggested by Dr. Bassi is that the nervous disturbance interferes with the due nutrition of the tissues in such a way as to give rise to the formation of toxic substances—probably ptomaines—which then set up acute degeneration of the parenchymatous elements similar to that which occurs in consequence of the action of certain poisonous substances such as phosphorus, or to that met with in some infectious diseases. In support of this view, he points out that Schule has found parenchymatous degeneration in persons dead from acute delirium, and that Zenker found hemorrhages in the pancreas in persons who had died suddenly; he refers also to some well-known facts concerning negroes in a state of slavery, and to the occasional occurrence of jaundice after fright. He hopes that these hints may induce medical officers of prisons and others to study, both clinically and anatomically, this by no means uninteresting or unimportant subject.—*The Lancet.*

THE OLD FAMILY DOCTOR.—Some of the older ones of us are like good old Dr. Kittridge, who had lived right among sick folks for five and thirty years, and had a library of five and thirty volumes bound up in his head at the end of that time. He knew the bigger part of all the families in a dozen miles of him—those that have the way of living through every thing, and the other set that have the trick of dying without any sort of reason for it. He knew the years when the fevers and dysenteries were in earnest, and when they were only making believe. He knew the folks that think they are dying as soon as they're sick, and those that never find out they're sick until they're dead. There are things he don't know, for they came in after his day, and he is very glad to send for those who do know them when he is at fault. But he knows the people in his neighborhood as all the science in the world can't know them, unless it takes time about it, and sees them grow up and grow old, and how the wear and tear of life comes to them.—*From the address of Pres. J. C. Sexton, M.D.*

